# How to assess performance in cycling: the multivariate nature of influencing factors and related indicators

**DOI:** 10.3389/fphys.2013.00116

**Published:** 2013-05-21

**Authors:** A. Margherita Castronovo, Silvia Conforto, Maurizio Schmid, Daniele Bibbo, Tommaso D'Alessio

**Affiliations:** Laboratory of Biomedical Engineering - Biolab3, Department of Engineering, University Roma TREVolterra, Rome, Italy

**Keywords:** cycling, performance monitoring, efficiency, effectiveness, fatigue, environmental variables, ergogenic aids

## Abstract

Finding an optimum for the cycling performance is not a trivial matter, since the literature shows the presence of many controversial aspects. In order to quantify different levels of performance, several indexes have been defined and used in many studies, reflecting variations in physiological and biomechanical factors. In particular, indexes such as Gross Efficiency (GE), Net Efficiency (NE) and Delta Efficiency (DE) have been referred to changes in metabolic efficiency (Eff_Met_), while the Indexes of Effectiveness (IE), defined over the complete crank revolution or over part of it, have been referred to variations in mechanical effectiveness (Eff_Mech_). All these indicators quantify the variations of different factors [i.e., muscle fibers type distribution, pedaling cadence, setup of the bicycle frame, muscular fatigue (MFat), environmental variables, ergogenic aids, psychological traits (Psych_Tr_)], which, moreover, show high mutual correlation. In the attempt of assessing cycling performance, most studies in the literature keep all these factors separated. This may bring to misleading results, leaving unanswered the question of how to improve cycling performance. This work provides an overview on the studies involving indexes and factors usually related to performance monitoring and assessment in cycling. In particular, in order to clarify all those aspects, the mutual interactions among these factors are highlighted, in view of a global performance assessment. Moreover, a proposal is presented advocating for a model-based approach that considers all factors mentioned in the survey, including the mutual interaction effects, for the definition of an objective function *E* representing the overall effectiveness of a training program in terms of both Eff_Met_ and Eff_Mech_.

## Introduction

The optimization of athletic performance is a matter of importance for all sports and, for a specific field such as cycling, is mainly related to the measurement of time or distance. Depending on the task (i.e., sprint, track time trial, endurance), cyclists are asked either to cover a fixed distance as fast as possible or to go as far as possible in a fixed amount of time. Then a performance improvement takes place when an athlete increases her/his previous results and hopefully manages to win the race. The improvement of performance is based on “efficacious” training, which aims at the development of motor strategies, including aspects such as energy expenditure and mechanical implementation.

From the analysis of the literature focusing on the development and implementation of such kind of trainings, different points of view emerge. For instance, most of the papers dealing with this issue use the terms (1) cycling efficiency (Coyle et al., 1991, 1992; Chavarren and Calbet, 1999; Hansen et al., 2002; Cannon et al., 2007; Korff et al., 2007, 2011; Leirdal and Ettema, 2011), (2) muscular efficiency (Whipp and Wasserman, 1969; Gaesser and Brooks, 1975; Neptune and Herzog, 1999; Zameziati et al., 2006; Hansen and Sjøgaard, 2007; Carpes et al., 2010), (3) mechanical efficiency (Umberger et al., 2006; Wakeling et al., 2010; Theurel et al., 2012). and (4) mechanical effectiveness (Eff_Mech_) (Zameziati et al., 2006; Korff et al., 2007; Ettema et al., 2009; Mornieux et al., 2010) with an almost equivalent meaning. This confusion is due to the fact that the terms “efficient,” “effective,” and “efficacious” share a common etymology but, since the real meaning is slightly different (Haynes, 1999), some clarifications are needed:

1. the term “*effective*” means that a method produces a decided, decisive, or desired effect (in the reality) (Efficiency, Effectiveness, Efficacy, Merriam-Webster's Collegiate Dictionary). It focuses on whether something either achieves the required objective or has a noticeable effect (e.g., “homework is an effective mean to let a student learn the topics of the lesson”);
2. the term “*efficient*” focuses on speed, ease, and convenience with which an objective is achieved: something is efficient if it works well without wasting time, money, or energy (Efficiency, Effectiveness, Efficacy, Merriam-Webster's Collegiate Dictionary). Something can be thus effective (i.e., it does the job) without being efficient;
3. ultimately, the term “*efficacious*” applies to things that are used for a certain purpose, such as medicines, or treatments: something is efficacious if it has the power of producing the desired effect (Efficiency, Effectiveness, Efficacy, Merriam-Webster's Collegiate Dictionary).

From now on in this paper we will refer to a cycling performance as the objective to be reached by using a specific training. This latter one can focus on a parsimonious use of the metabolic resources, thus aiming at the metabolic efficiency (Eff_Met_), and/or can deal with the improvement of the cycling gesture, thus improving Eff_Mech_ as well. The crucial point is that, in order to achieve a desired level of performance, there are different ways to develop and implement motor strategies, which depend, in turn, on the optimization of either Eff_Met_ or Eff_Mech_ (Neptune and Herzog, 1999; Korff et al., 2007; Sarre and Lepers, 2007; Mornieux et al., 2010). These two quantities are influenced from, and mutually connected to, several factors, even different in nature, that have not been yet completely investigated, and whose cross-effects are still far from being fully understood. Among these factors it is worth citing the following: muscle fibers distribution (Staron and Pette, 1986; Coyle et al., 1991, 1992; Ahlquist et al., 1992; Hansen et al., 2002; Umberger et al., 2006; Hansen and Sjøgaard, 2007), pedaling cadence (Chavarren and Calbet, 1999; Neptune and Herzog, 1999; Neptune and Hull, 1999; MacIntosh et al., 2000; Lucía et al., 2001; Hansen et al., 2002; Umberger et al., 2006; Bieuzen et al., 2007; Hansen and Sjøgaard, 2007; Mornieux et al., 2008; Abbiss et al., 2009; Ettema et al., 2009; Vercruyssen and Brisswalter, 2010; Leirdal and Ettema, 2011), biomechanical characteristics (Davis and Hull, 1981; Coyle et al., 1991; Neptune and Herzog, 2000; Bibbo et al., 2006; Cannon et al., 2007; Korff et al., 2007, 2011; Sarre and Lepers, 2007; Van Sickle and Hull, 2007; Mornieux et al., 2008, 2010; Carpes et al., 2010; Wakeling et al., 2010; Theurel et al., 2012; Romanov, 2008), ergogenic factors, which include dietary supplements and psychological strategies (Morgan, 1973; Morgan et al., 1973; Foster et al., 1994; Morgan, 1985; Dietary Supplement Health, 1994; Ulmer, 1996; Garcin et al., 1998; Berger et al., 1999; Raglin, 2001; Williamson et al., 2001; Albertus et al., 2005; Williams, 2004, 2005; MacRae and Mefferd, 2006; Tucker et al., 2006; Bishop, 2010; Waterhouse et al., 2010) and, last but not least, muscular fatigue (MFat) (Coast and Welch, 1985; Neptune and Hull, 1999; Lepers et al., 2002; Abbiss and Laursen, 2005; Theurel and Leperd, 2008; Bini et al., 2010; Theurel et al., 2012).

When focusing on Eff_Met_, muscle fiber type, pedaling cadence, oxygen consumption, ergogenic aids and training can be listed as influencing factors. When shifting the attention to Eff_Mech_, some factors are maintained (pedaling cadence and training), and new ones are introduced (e.g., power output and mechanical setup of the bicycle). Moreover, the contribution of MFat cannot be underestimated, since it is a disturbing element for both efficiency and effectiveness leading to a general decline of performance. Muscular fatigue, due to its physiological genesis, is directly linked to dietary supplements and psychological factors, which, in some way, influence the performance of athletes, either beneficially or detrimentally.

Most of the studies in literature keep Eff_Met_ and Eff_Mech_ separated, and they use heterogeneous parameters (e.g. cycling velocity, oxygen consumption, neuromuscular efficiency, energy expenditure, or force exertion) as performance indicators. These are generally associated with the metabolic cost of the task, the biomechanics of the gesture, and the time and/or the race distance. In this way, the most efficacious treatment, intended as the optimum with respect to an objective function depending on both Eff_Met_ and Eff_Mech_, cannot be directly determined, and the general question of how to improve the cycling performance cannot be answered.

This paper wants to make a proposal in the direction of finding an optimum for the function evaluating the efficacy of training, by revising the literature of the field and highlighting those controversial aspects still present. A review of the indexes used as quantitative estimators of performance in cycling is presented as well, together with a report of physiological, psychological and biomechanical factors influencing cycling gesture and its correlates. Attention is devoted to the analysis of cross-effects regarding all these parameters, and to the way these indexes and factors are correlated with the motor strategies leading to that performance, more than assessing the performance *per se*. A computational model is then evoked as a prospective solution for integrating all the factors that affect the performance assessment in this field, possibly overcoming the difficulty to take them into account simultaneously in experimental protocols.

For the authors, the development of a computational model, trying to overcome the difficulties associated with setting up an experimental protocol for multifactorial analysis, is considered as a possible implementation of the multivariate analysis of the performance. This approach is justified by the outcomes of the literature review presented in this work.

## Indexes for cycling performance assessment

### Metabolic indexes

Endurance sports rely on aerobic metabolism for energy demand. Thus, the main factors related to oxygen consumption, i.e., VO_2_ and VO_2Max_, are assumed as determinants of endurance exercise performance. VO_2_ is a measure of the O_2_ volume used in the energy conversion process into ATP molecules, needed by the muscles to continue working during exercise. VO_2Max_ is related to the maximum exercise intensity that a subject can withstand without further increases in VO_2_, and is often used as an indicator of performance *per se* (Cerretelli and Di Prampero, 1987).

The concept of Eff_met_, defined as the ratio between the exerted work and the expended energy, is based on the assessment of VO_2_ kinetics. This ratio has been quantified by indexes such as “Gross Efficiency” (GE) (Whipp and Wasserman, 1969), “Net Efficiency” (NE) (Gaesser and Brooks, 1975), and “Delta Efficiency” (DE) (Coyle et al., 1992; Zameziati et al., 2006). GE represents the overall metabolic expenditure and is expressed by the following equation (Equation 1).

(1) $$GE(\%)=100\times \left(\frac{W_{\text{ext}}}{VO_{2}\times \kappa }\right)$$

where W_ext_ is the accomplished work per minute, VO_2_ (l·s^−1^) is the oxygen consumption at steady state and κ = 20.9 kJl^−1^ is the energetic equivalent for O_2_.

NE is similar to GE, from which it differs because the contribution of oxygen consumption at rest is subtracted (Equation 2).

(2) $$NE(\%)=100\times \left(\frac{W_{\text{ext}}}{(VO_{2}-VO_{\text{2rest}})\times \kappa }\right)$$

Finally, DE does not take into account the influence of those metabolic processes that do not contribute to the accomplished work, and thus is not an integral parameter; rather it represents an incremental ratio measure (Equation 3).

(3) $$DE=\frac{\Delta W_{\text{ext}}}{\Delta VO_{2}}$$

For this reason it has been considered as a more viable performance indicator than both GE and NE (Gaesser and Brooks, 1975; Coyle et al., 1992; Zameziati et al., 2006).

### Mechanical indexes

**Figure 1:**
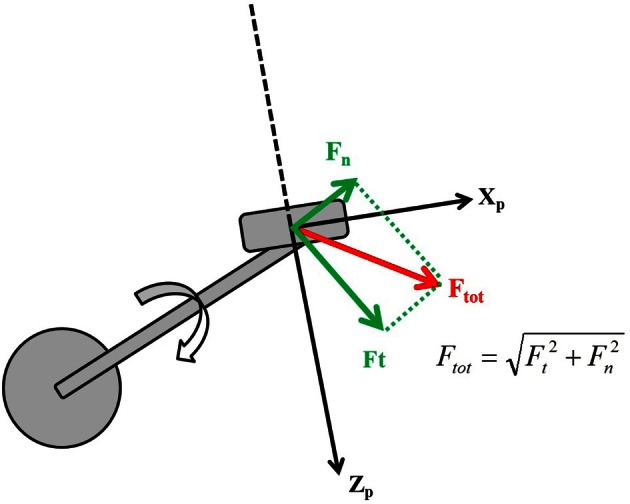
**Forces applied to the pedal in the pedal reference system {*X*_*p*_, *Z*_*p*_}**.

The Eff_mech_ is mainly related to the way forces are applied on the pedal (Coyle et al., 1991; Zameziati et al., 2006; Mornieux et al., 2010; Romanov, 2008). It can be ranked in terms of ratio between the *useful* component of force that is the one tangential to the crank (*F*_t_) and the overall one (*F*_tot_), applied by the foot to the pedal load surface (Bibbo et al., 2008) (see Figure 1).

**Figure 2:**
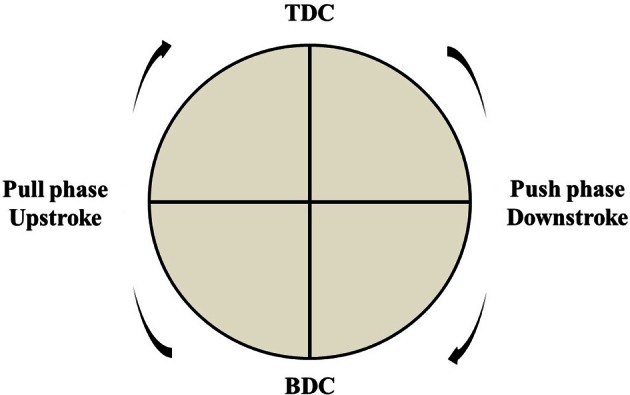
**Pedal cycle: from top dead center (TDC) to bottom dead center (BDC) (push phase) and again from BDC to TDC (pull phase)**.

This ratio has been expressed through different indicators [that share the same notation “Index of Efficiency,” (IE)], expressed as a percentage evaluated over the pedal cycle (see Figure 2).

In particular, IE_360°_ (Equation 4) is defined over the entire pedal cycle (Davis and Hull, 1981), while IE_180°Desc_ (Equation 5) and IE_180°Asc_ (Equation 6) are defined over the descending (i.e., downstroke) and the ascending (i.e., upstroke) phases, respectively (Coyle et al., 1991; Zameziati et al., 2006).

(4) $$IE_{360^{{}^{\circ}}}(\%)=\frac{\int_{0}^{2\pi }F_{t}(\vartheta )\times d\vartheta }{\int_{0}^{2\pi }F_{\text{tot}}(\vartheta )\times d\vartheta }\times 100$$

(5) $$IE_{180^{{}^{\circ}}\text{Desc}}(\%)=\frac{\int_{0}^{\pi }F_{t}(\vartheta )\times d\vartheta }{\int_{0}^{\pi }F_{\text{tot}}(\vartheta )\times d\vartheta }\times 100$$

(6) $$IE_{180^{{}^{\circ}}\text{Asc}}(\%)=\frac{\int_{\pi }^{2\pi }F_{t}(\vartheta )\times d\vartheta }{\int_{\pi }^{2\pi }F_{\text{tot}}(\vartheta )\times d\vartheta }\times 100$$

## Factors related to cycling performance

### Muscle fibers

Since muscle fibers produce energy from ATP (Kushmerick, 1983; Staron and Pette, 1986; Coyle et al., 1991; Ahlquist et al., 1992; Hansen et al., 2002; Umberger et al., 2006), they are directly connected to cycling efficiency. The way the ATP reaction occurs, depending on the myosin heavy chain forms (MHC-I or II) (Staron and Pette, 1986), allows a classification of the human skeletal muscle fibers into the following types: (i) *slow twitch* (ST) or type I fibers, characterized by slow firing rates and a good resistance to MFat; (ii) *fatigue-resistant* (FR) or type IIA fibers, resistant to MFat and able to produce high force levels; (iii) *fast twitch* (FT) or type IIB fibers, producing short-time peaks of force and not FR.

The distribution of the muscle fibers can influence the cycling performance in terms of VO_2_ response, which can be quantified by Eff_met_. This distribution, in the muscles, is quite heterogeneous, and the percentage of ST and FT fibers heavily influences the indexes assessing the performance, as confirmed also by quantitative models (Coyle et al., 1992; Umberger et al., 2006).

### Pedaling cadence

The existence of an optimal Pedaling Cadence (PC) has not been demonstrated yet, because there are some PCs optimizing the Eff_Met_ and other ones improving the neuromuscular one (Faria et al., 2005).

With respect to the neuromuscular efficiency, the optimum PC is the one minimizing the overall muscle activation and the MFat (Abbiss et al., 2009; Theurel et al., 2012). Neptune and Herzog (2000) have demonstrated that a 90 rpm PC asks the muscles for the minimum force levels, while 93.5 rpm (Bieuzen et al., 2007) is the value that maximizes the neuromuscular efficiency in well-trained cyclists. On the contrary, higher PCs are responsible for increased metabolic costs (Chavarren and Calbet, 1999).

It was demonstrated that triathletes, during prolonged exercises, tend to choose a PC close to the energetically optimal one, as determined analytically based on the relation between cadence and oxygen uptake variations (Brisswalter et al., 2002), by changing the muscle activity pattern, and this may explain the shift toward higher PCs (Brisswalter and Hausswirth, 2000). The energetically optimal PC has been found at around 63.5 rpm in (Bieuzen et al., 2007), at about 50 rpm in (Chavarren and Calbet, 1999; Zameziati et al., 2006), and in the range 90–105 rpm for professional riders in (Lucía et al., 2001). These rather contrasting results need to be mingled with the values reported by Vercruyssen and Brisswalter (2010), who showed that the energetically optimal PC falls in a range (i.e., 55–65 rpm) different from the freely chosen PC range (i.e., 80–95 rpm). Marsh and Martin (1998) tried to associate the preferred pedaling cadence to the rate of perceived exertion in professional cyclists, runners and non-cyclists. They found out that, irrespective of Borg's scale (Borg, 1970), the pedaling rates minimizing Ratings for Perceived Exertion (RPE) were lower than the selected preferred ones for all subjects, concluding that the changes in RPE are not critical for cadence selection during submaximal cycling.

### Biomechanics

Several biomechanical factors affect the pedaling performance, first of all the Pedaling Technique (PT). It can be regarded as the way the cyclist pedals, and thus it includes the type of pedaling gesture (mash, circular or triangular; right vs. left leg dominance), the geometry of the bike frame, the saddle position and its height, the crank length. These factors heavily affect the way the main muscles involved in the cycling task are used, and have been extensively investigated in literature, both studying the specific contribution of each of them (Jorge and Hull, 1986; Bibbo et al., 2006) and analyzing how they work in synergy (De Marchis et al., 2012). Muscles are activated in different phases of the crank cycle, according to their principal functions (Bibbo et al., 2006), and, while mono-articular muscles are mainly power producers, the bi-articular ones act mainly to transfer energy between joints during the pedal revolution (Van Ingen Schenau et al., 1992).

Since the force demands drive the energy requests, it appears that reducing muscular forces at a given power output may improve the performance. One way to decrease the force developed by the ankle plantar-flexors is to move the foot anteriorly on the pedal, thus balancing the moment about the ankle caused by the reaction force of the pedal. This force reduction could be translated into a better Eff_met_ (Coyle, 1995; Van Sickle and Hull, 2007), even if the relationship between GE and IE is not direct. The mechanical energy produced by multiple muscles is, in fact, transferred via the body segments to the crank and this could make Eff_mech_ not resulting in an improved Eff_met_.

Several studies assessed the influence of technical factors on performance: the pedal type (Mornieux et al., 2008), the rider position and the pedaling technique (Korff et al., 2011; Romanov, 2008), different chain rings (Kautz, 1994), the inclination of the seat (Leirdal and Ettema, 2011). Shoe-pedal interfaces do not influence the pedaling pattern and VO_2_ during submaximal cycling, but during uphill cycling, where maximal power is required, wearing of clip-less pedals may be advantageous (Mornieux et al., 2008). Regarding the position of the rider on the seat, the “Pose” method (Romanov, 2008) was introduced to define a PT improving Eff_met_: this method benefits from the gravitational contribution to pedal power, and corresponds to a lower seat height and a more upright body position. These factors lead to a GE increase when compared to subject's preferred bicycle position, even if aerodynamic conditions are disregarded. The combined effect of changing bicycle setup and PT showed no effect on GE, and only small effects on pedaling mechanics (Leirdal and Ettema, 2011). Different chain rings affect the angular velocity of the crank-arm and, in turn, the mechanical work produced to move the legs (Kautz, 1994).

### Muscular fatigue

The onset of muscular fatigue (MFat), intended as an exercise-induced reduction of voluntary force (Coast and Welch, 1985; Lepers et al., 2002), is an important factor affecting cycling performance. Different cause-and-effect models have been developed to address this topic without, however, fully explaining the phenomenon (Abbiss and Laursen, 2005). The reduction in force levels, concomitant with the increasing exercise duration, leads to a drop of the muscle activation level at the end of the exercise, because of an impairment of contractile properties and of an alteration of both excitability and central drive (Lepers et al., 2002; Castronovo et al., 2012). During prolonged cycling, changes in PT can influence the occurrence of MFat and also the energetic demand. In contrast with studies showing occurrence of MFat after prolonged exercises (Coast and Welch, 1985; Lepers et al., 2002), some authors have reported its onset just after 15 min of exercise, irrespective of PT (Theurel and Leperd, 2008). The influence of MFat on performance can be better evaluated during cycling until voluntary exhaustion, by introducing also biomechanical data, such as the net joint moment distribution or the joint forces and kinematics (Bini et al., 2010). For example, the ankle joint contribution to the net joint moment decreases with increasing MFat (Lepers et al., 2002).

### Ergogenic aids

#### Dietary supplements

With the term dietary supplement (Diet) it is intended “*any product taken by the mouth in addition to common foods, which has been proposed to have a performance-enhancing effect*” (Bishop, 2010). Several studies have been dedicated to the evaluation of the improvement of cycling performance via dietary supplements (Williams, 2004, 2005; MacRae and Mefferd, 2006). Many athletes need a correct nourishment to improve their physiological performance, preventing them to use pharmacological agents as steroids or amphetamines (Williams, 2004). The Dietary Supplement Health and Education Act (DSHEA) in (1994) clarified the dietary supplements that can be assumed by athletes, helping them in improving their sport performance: vitamins, minerals, amino acids, herbs, and botanicals, metabolic constituents. For example it has been demonstrated that B vitamins are necessary for physiological mechanisms as carbohydrate and fats processing for ATP production, but also C and E vitamins act as antioxidants preventing cellular and subcellular damages during exercise training. Mineral, instead, have been demonstrated to be unnecessary for athletes with an already well-balanced nutrition, as well as vitamin C supplementation. Vitamin E enhances the oxygen utilization during exercise at higher altitude levels but it has been revealed ineffective during cycling at sea level condition (Williams, 2004, 2005). Moreover, the antioxidant supplementation combined with flavonoids as Quercetin (FRS), which can be found in blueberries, cranberries, crowberries, and grapes but also in red onions or apples, has been found to improve average power and % peak power during 30 km cycling trials, without significant amelioration of % HR max or VO_2_ (MacRae and Mefferd, 2006). Also caffeine effects have been evaluated in several studies focused on cycling activities (Jeukendrup and Martin, 2001; Foad et al., 2008). In fact, it has been reported that the use of caffeine improves the performance and endurance capacity when it does not exceed the threshold concentration defined by the International Olympic Committee (12 mg/l) (Jeukendrup and Martin, 2001; Foad et al., 2008).

#### Psychological traits

The performance of an athlete may change also in relation of his/her perceived exertion, which “integrates various information, including many signals elicited from the peripheral working muscles and joints, from the central cardiovascular and respiratory functions, and from the central nervous system” (Borg, 1982). In order to assess the perceived exertion, the scale developed by Borg (1970), referred to as RPE scale (Borg, 1970, 1982), has been used in different application fields, including cycling.

O'Sullivan in her review on perceived exertion mentioned all the physiological and psychological variables which showed a correlation with RPE (O'Sullivan, 1984), and several studies have considered the RPE in relation to cycling exercises (Borg and Linderholm, 1967; Skinner et al., 1969; Noble et al., 1973; Garcin et al., 1998; Tucker et al., 2006). In particular, the variance that could not be explained with physiological variables (around 33%), such as HR, force produced, ventilatory and oxygen response or gender, was estimated as coming from psychological factors (Morgan, 1973). In fact, the effect that a particular mental status can have on athletes' performance is not to be underestimated: subjects that are depressed, neurotic or anxious tend to process information related to muscular work more unlikely than individuals without these disturbances (Morgan, 1973). As of now, the focus of sports medicine is upon maintaining the physical health of the athletes, and this means including psychological variables as well, because of their impact on performance and general status (Raglin, 2001). For example, well-trained athletes resort to “*pacing strategies*” to optimize performance during cycling or running, which is a subjective way to use and distribute their own sustainable power output (i.e., effort) in a wise way during the overall duration of the race (Foster et al., 1994). One remarkable work including psychological aspects predicting performances of athletes focused on a Mental Head Model (MHM) (Morgan, 1985) and thus included psychological traits (Psych_Tr_) as neuroticism, confusion, anxiety stress and fatigue, which are likely to invalidate the performance of the athlete (Morgan, 1973; Raglin, 2001). Changes in mood upon athletic performance in response to high intensity training or to the introduction of music during training have also been evaluated by other studies (Berger et al., 1999; Waterhouse et al., 2010). For example, it has been demonstrated that fast music tempo positively influences the performance acting on motivation and distracting effects compared to slow music during low and moderate intensity cycling exercise (Waterhouse et al., 2010). Another topic to be mentioned concerns the use of acting drugs or hypnosis, which may result in an alteration of psychological responses (Albertus et al., 2005). Some studies are focused upon hypnotic manipulation of effort, and, in particular, on the physiological responses following this psychological treatment during cycling. Morgan et al. (1973) evaluated metabolic responses during various and different hypnotic suggestions either at a constant workload and PC and they did find out that hypnosis modified physiological outcomes. As a matter of fact, some subjects thought that the duration of the exercise, and not its intensity, was reduced under hypnosis; the HR was higher during suggestion of heavy work and lower during the suggestion of light work, so following individual suggestion, even if the workload did not actually changed at all. Williamson et al. (2001) evaluated cardiovascular and cerebral responses, in an attempt to separate the descending signals originating from the brain in response to afferent inputs coming from peripheral pathways, and thus to determine whether cortical structures involved in cardiovascular modulation are activated during hypnotic suggestion of downhill or uphill cycling. The hypnotic suggestion, according to the authors, should not involve central commands, which rely on a feed-forward mechanism of activation of both motor and cardiovascular centers. The authors concluded that cerebral cortical structures (right insular cortex and right thalamic region), during hypnosis, are activated by an increased sense of effort, thus reflecting an augmented cardiovascular response; this is not paralleled by a corresponding reduction of their activation, when a decreased sense of effort is generated.

Including these aspects into a global performance assessment is not a trivial matter since they are mostly based on scores lying on different scales [i.e., Borg's Scale (Borg, 1982), Berber Suggestibility Scale (BSS) (Barber, 1969), Eysenck Personality Inventory (Eysenck and Eysenck, 1963), Spielberger's state-trait inventory (Spielberger et al., 1969), Somatic Perception Questionnaire (Landry and Stern, 1971), Lubin's Depression Adjective Checklist (Lubin, 1967)]. Moreover, studies on the psychological influence on biomechanical variables, intended as index of effectiveness of produced forces, are still lacking in literature and an integration of these three aspects is really needed.

#### Environmental variables

In the cycling field, as it happens in many motor tasks, the influence of the environment on the execution of the gesture has to be taken into account. This aspect can be explained by considering if and how the performance of a cyclist can be affected by stimuli coming from the environment (Kay et al., 1999; Marsh and Sleivert, 1999; O'Brien and O'Conner, 2000; Waterhouse et al., 2010). Biofeedback techniques (Sanderson and Cavanagh, 1990; Sveistrup, 2004; Hasson et al., 2008) aim at improving the pedaling performance by stimulating different sensorial channels of the athlete: through a set of recording devices, processing algorithms and graphical user interfaces (GUIs) it is possible to extract information to be presented in real-time to the athlete (Sanderson, 1987). In this way, biomechanical or metabolic information return to the subject's brain as a feedback. Thus, the peripheral input and the way it is integrated in the central paths influences the subsequent plan of exercise intensity (pacing strategy) (Ulmer, 1996). Different representations are used to provide information to the athlete, and some studies in the literature focus on the use of visualization techniques (Aris et al., 2005). The aim of letting cyclists learn to pull up on the pedal and thus increase Eff_Mech_, has driven some authors to present visual feedback to the riders in rather different ways (Sanderson, 1987; Mornieux et al., 2010; Bibbo et al., 2012). The above-mentioned studies reported a significant variation of the pedaling gesture with the use of visual feedback confirming the hypothesis that, independently of the particular rendering scheme, the use of biofeedback allows riders to improve Eff_Mech_. No evidence exists concerning the improvement of Eff_Met_ in biofeedback-based training, evidencing the lack of convergence on a global assessment of cycling performance.

The place where the cyclist lives and practices is strictly connected to the cycling performance. In order to maximize adaptations to altitude and minimizing its influence to training, a hybrid approach, named Live High Train Low (LHTL) has been developed, consisting in living at moderate altitudes and training at sea level or low altitudes (Hahn and Gore, 2001). An exposure to moderate altitude seems to enhance sport performance at sea level ground since the benefit of reduced aerodynamic drag overcomes the decrease in maximum aerobic power, estimated as VO_2Max_. Training at moderate altitudes, thus breathing with higher levels of oxygen than those experienced in the lifetime, determines an increase in aerobic power, more than a higher aerobic capacity. Other studies have demonstrated that aerobic exercise performance decreases upon ascent to altitude whereas anaerobic performance remains unchanged (Burtscher et al., 2006). On the other hand, training in a hyperoxic environment may lead to higher training intensities, which result in a significant improvement in maximal steady state power output (Morris et al., 2000). The exposure to altitude has been demonstrated to be related also to time trial performance: there was no change in the 5-min cycling performance but the 50-min cycling performance improved after 45 h of altitude acclimatization (Burtscher et al., 2006).

## Mutual interactions among factors affecting the performance

A complete characterization of athletes' performance could be misleading, if the factors mentioned above are kept separated. Focusing on single factors may determine results different from those obtained when all the factors are considered together.

PC is affected simultaneously by several factors, such as the power output (Coast and Welch, 1985) and the changes in the fiber muscle recruitment pattern (Ahlquist et al., 1992; Umberger et al., 2006), but also by the rider's skill (Umberger et al., 2003) and the workload (Coast and Welch, 1985; Hansen et al., 2002; Foss and Hallen, 2004). No influence is reported when considering the relationship between VO_2_ (and its kinetics) and PC, irrespectively of fiber type distribution. The prevailing presence of MHC-I is related to high pedaling rates but not to maximum values of GE (Hansen et al., 2002). In particular, the correlation between MHC-I and GE is positive when subjects pedal at preset pedal rates, and becomes negative when a freely PC is chosen (Chavarren and Calbet, 1999; Hansen et al., 2002; Zameziati et al., 2006). Musculoskeletal models and computer simulations confirmed those experimental values (Seabury et al., 1977; Coast and Welch, 1985; Neptune and Hull, 1999; Umberger et al., 2003, 2006). It has also been reported that a low PC (around 50 rpm), for the same metabolic cost, causes augmented muscular forces when compared to higher PCs (Ahlquist et al., 1992). Supporting this hypothesis, the required level of force was found as the factor determining the decrease of PC during an endurance exercise (Coast and Welch, 1985; Lepers et al., 2000). This fall in cadence, concomitant with an increase in exercise duration and occurrence of muscle fatigue, is interpreted as an adaptation of the movement pattern in order to minimize the energy cost rather than the neuromuscular one. If, instead, we focus on submaximal workloads, a unique PC was found to minimally activate the muscles (MacIntosh et al., 2000). During submaximal exercises with constant PCs, IE was found to increase, especially in the downstroke phase, resulting as an important factor for changes in Eff_met_ (Zameziati et al., 2006).

PC affects, on the other hand, the muscular activations and patterns, making thus impossible to estimate the timing of muscular internal forces from the forces applied on the pedal. Muscle power, as soon as the cadence increases, is generated at a later crank angle, making the choice of the preferred PC dictated not only by metabolic costs. Higher PCs increase the inertial non-muscular component of the pedal forces, which is closely related to fluctuations of the kinetic energy.

In this perspective, PC seems to affect both GE and IE but no causal relationship between the two indexes emerges: inertial forces, in fact, do not have any metabolic cost, and the increase of cycling economy cannot be linked to the decrease of IE.

Moreover, PT is a further controversial aspect for performance evaluation. Changing PT modifies the number of muscles involved and their activation timing, and may lead to a variation (that may be detrimental) of some physiological variables: for example, switching to a dorsi-flexed PT increases metabolic costs (Zameziati et al., 2006). The activity of each muscle involved in the task depends on the mechanical demand (Bibbo et al., 2006; Wakeling et al., 2010), which drives the generation of forces applied to the pedals. When forces are not applied correctly, as it can be monitored by the variations of IE_360°_, IE_180°Desc_, IE_180°Asc_, inefficient muscular work is produced (Neptune and Herzog, 1999; Zameziati et al., 2006; Mornieux et al., 2010). These findings are, however, in contrast with those showing an increase of IE with a concomitant worsening of metabolic behavior under different PTs (Korff et al., 2007). The latter results are supported and well explained by the analysis of the pull-up action on Eff_mech_ and Eff_met_ (Mornieux et al., 2008): this action is thought to be responsible for an augmented Eff_mech_ during the upstroke phase (which causes a higher IE_360°_), but it is also associated with an increased muscular work and co-activations, leading to augmented VO_2_ (Mornieux et al., 2008). This has been correlated to the training volume affecting the pedaling pattern: any induced alteration, such as an active pulling-up action, could impair the physiological response. Different is the case of non-experienced cyclists who, not exhibiting an intrinsic pedaling pattern due to the absence of previous training, can improve Eff_mech_ without altering VO_2_ (Mornieux et al., 2010).

All these counteracting aspects, when considered together, lead to a wide range of PTs, each with its optimal PC, eliciting similar levels of Eff_met_, and these findings are at odds with the hypothesis of having just one optimal PC, as discussed before (Korff et al., 2007).

## A perspective model

What may thus be considered as the best way to evaluate a cyclist's performance?

So far, the effectiveness of the training in cycling and, in turn, the goodness of the performance, has been assessed in several ways, mainly focusing on physiological factors (through the Eff_met_) or on the pedaling technique and its correlates (through the Eff_mech_). Each of these two approaches takes into account muscular variables, pedaling cadence, biomechanical factors, environmental variables, and also the occurrence of MFat. These factors are often analyzed separated from each other or, in some cases, considered as sub-sets by looking at the effects of one or two of them on both efficiency and effectiveness. We are under the impression that this way does not lead to a complete evaluation of the performance. If e.g., we want to find the pair of variables {cadence PC, seat height H_S_} that optimizes the performance, by using the Eff_Met_ as the objective function, we may probably find an optimum set {PC_eff_, H_S_} which is different from the one obtained by using Eff_Mech_ as the objective function. In addition to that, these input variables interact with other status variables. For example, repetitive training with a changed H_S_ will determine variations in muscular timing patterns so affecting the muscle fiber distribution. This change in distribution will modify in a recursive manner the shape of the objective function taken into consideration.

In an attempt to suggest a solution that could help in highlighting the relationships between the different parameters, we propose here to use a computational model. The idea of using models to optimize performance is not new in literature (Martin et al., 1988; Neptune and Hull, 1988; Olds et al., 1993; Swain, 1997; Jeukendrup and Martin, 2001; Olds, 2001; Abbiss and Laursen, 2005), because the mathematics allows to simplify the problem by reducing it to an expression of the type *y* = *f*(*x*), where *x* represents a combination of biomechanical and/or physiological parameters and *y* is a performance variable (Olds, 2001). The studies published in literature used different combinations of input variables, and defined different performance variables. Some of those based the performance on the effect of physical variables on the athlete (i.e., altitude, aerodynamic setup, altitude variations upon energy supply) (Olds, 2001), others tried to predict the power output with respect to aerodynamic resistance, wheel rotation, rolling resistance, and changes in potential and kinetic energy (Martin et al., 1988). Jeukendrup et al., starting from the work of Martin et al. (1988), synthesized the factors that can influence cycling performance and divided them into internal (i.e., training, altitude training, carbohydrate and caffeine) and external factors (i.e., body weight, body position, clothing, bicycle, and wheels) (Jeukendrup and Martin, 2001). A model of this kind, even if considering several factors among biomechanical and physiological, lacks of integration of information about muscular status and MFat or psychological variables. Neptune and Hull also developed a model and an optimization framework to simulate a pedaling exercise at submaximal power, with a main focus on kinetic, kinematic and activation timing quantities, but without considering all the physiological correlates (Neptune and Hull, 1988). A model to optimize cycling performance varying power on uphill and windy conditions was also developed by Swain and pointed attention on time saving and its relation to VO_2_ variations, but without considering the technical frame of the bike or the muscular status (Swain, 1997).

None of these models integrates all the factors that affect the performance, and so limits the multivariate analysis of the phenomenon. In this review, it is suggested to put together design specifications derived by experimental outcomes, with mathematical techniques already developed to solve multi-variables optimization problems. Typically, solving an optimization problem means locating the value set that corresponds to the maximum value of the objective function. If the objective function is the overall effectiveness (E) of a training program, we may consider the following equations:

$$\left\{ \begin{array}{l} E=f\text{​}({\text{Eff}}_{\text{Met}},{\text{ Eff}}_{\text{Mech}})\\ {\text{Eff}}_{\text{Met}}=g(m_{\text{prop}},\text{ PC},{\text{ Biomech}}_{\text{set}},\text{ MFat},\text{ Diet},{\text{ Psych}}_{\text{Tr}})\\ {\text{Eff}}_{\text{Mech}}=h({\text{Biomech}}_{\text{set}},\;m_{\text{prop}},{\text{ Envir}}_{\text{var}}) \end{array}\right.$$

where the underlying hypothesis is that *E* is a function of both Eff_Mech_ and Eff_Met_. According to our literature survey, we may consider that Eff_Mech_ is only directly dependent on biomechanical configuration Biomech_set_, muscular properties m_prop_ and environmental variables Envir_var_, while Eff_Met_ adds to these factors pedaling cadence PC and MFat, and Diet and Psych_Tr_ as well. In this simplified scheme, the mutual interactions are not highlighted, but the interdependency can be listed as well according to the following equations that summarize the relations found in the literature:

$$\left\{ \begin{array}{l} \text{MFat}=u(m_{\text{prop}},\text{ Diet},{\text{ Envir}}_{var},{\text{ Psych}}_{\text{Tr}},{\text{ Biomech}}_{\text{set}},\text{ PC})\\ m_{\text{prop}}=v(\text{MFat},\text{ Diet})\\ {\text{Biomech}}_{\text{set}}=w(m_{\text{prop}},{\text{ Envir}}_{\text{var}}) \end{array}\right.$$

where the fatigue variable depends on the specific biomechanical configuration and may depend on the environmental variables; the muscular properties, and their activation patterns, are dependent on fatigue, and their change may determine a variation in the biomechanical configuration that is used. Also the Psych_Tr_, as we said before, have an influence on physiological performance and thus on Eff_Met_ and may change the MFat variable, but since the connection with the mechanical outcomes is lacking, a computational model could help estimate this relation.

By applying mathematical laws to express the mutual interactions and recursions, we are confident that it could be possible to find a solution for the maximization of the effectiveness function *E*, by considering all its dependencies on the listed variables. Moreover, by using suitable instrumentation (Bibbo et al., 2008, 2012) and objective processing techniques, respectively, to measure the biomechanical properties and to estimate the activation patterns in terms of amplitude (D'Alessio and Conforto, 2001) and timing (Bonato et al., 1998; Vannozzi et al., 2010; Severini et al., 2012) and spectral characteristics (Conforto and D'Alessio, 1999), also the model validation appears as a feasible operation.

Further researches are needed in order to define the mathematical relationships explaining mutual interactions and thus, ultimately, defining the model. The model, in fact, could be also used to investigate the variation of muscular activation strategies linked with performance changes (De Marchis et al., 2013). In our opinion, the model is one of the few viable solutions for understanding the multiple factors affecting a performance and thus, in perspective, for the development of training techniques based on the reported scientific evidences.

### Conflict of interest statement

The authors declare that the research was conducted in the absence of any commercial or financial relationships that could be construed as a potential conflict of interest.
